# The Fragility Index in a Cohort of Pediatric Randomized Controlled Trials

**DOI:** 10.3390/jcm6080079

**Published:** 2017-08-14

**Authors:** Travis J. Matics, Nadia Khan, Priti Jani, Jason M. Kane

**Affiliations:** 1 Department of Pediatrics, Section of Critical Care Medicine, The University of Chicago Medicine, Chicago, IL 60637, USA; travis.matics@uchospitals.edu (T.J.M.); nkhan@bsd.uchicago.edu (N.K.); priti.jani@uchospitals.edu (P.J.); 2 Center for Healthcare Delivery Science and Innovation, The University of Chicago Medicine, Chicago, IL 60637, USA

**Keywords:** randomized controlled trials, research methodology, statistics, pediatrics, clinical trials

## Abstract

Data suggest inadequacy of common statistical techniques for reporting outcomes in clinical trials. The Fragility Index can measure how many events the statistical significance hinges on, and may facilitate better interpretation of trial results. This study aimed to assess the Fragility Index in pediatric randomized controlled trials (RCTs) with statistically significant findings published in high-quality medical journals. A Fragility Index was calculated on included trials with dichotomous positive outcomes. Analysis of the relationship between trial characteristics and the Fragility Index was performed. Of the 429 abstracts screened, 17 met the inclusion criteria and underwent analysis. The median Fragility Index was 7 with an interquartile range of 2–11. In 41% of the studies, the number of patients lost to follow-up or withdrawn prior to analysis was equal to or greater than the Fragility Index. There was no correlation between the RCT sample size and the Fragility Index (*r* = 0.249, *p* = 0.335) nor the event group size and the Fragility Index (*r* = 0.250, *p* = 0.334). There was a strong negative correlation between the original *p*-value and the Fragility Index (*r* = −0.700, *p* = 0.002). The Fragility Index is a calculated metric that may assist in applying clinical relevance to statistically significant outcomes in pediatric randomized controlled trials with dichotomous outcomes.

## 1. Introduction

Although often deemed the gold standard for evidence-based medicine, well-designed randomized controlled trials (RCTs) in pediatric critical care medicine are sparse. Given their relative rarity, the accurate interpretation of results from pediatric critical care RCTs is paramount in ensuring that high-risk clinical decisions and interventions in the intensive care unit (ICU) are supported by the best available evidence. Ideally, a clinical trial suitable for publication in a high-quality medical journal must be well designed, with an appropriate sample size and power calculations explicitly stated, allowing for accurate interpretation and application of the results. Traditionally, *p*-values have been used to denote the statistical significance of RCT results, but not without significant limitation and subsequent criticism [1,2,3,4]. Additionally, *p*-values are often inappropriately applied, misinterpreted, and erroneously reported [5]. As a result, many high-quality journals now refer authors to the Consolidated Standards of Reporting Trials (CONSORT) statement which encourages the reporting of both the estimated effect size and its precision through the use of *p*-values and confidence intervals [6]. The addition of the confidence interval calculation allows clinicians to not only ascertain whether there is a significant difference between the two experimental groups, but also the magnitude of that difference [7]. However, even with a *p*-value and a confidence interval, the clinician cannot immediately discern how likely the study, if repeated, would yield a different and potentially conflicting result. 

The Fragility Index was developed as a novel metric to further assess the quality of statistically significant results and assist with the interpretation and clinical applicability of RCT findings [8]. In its most basic terms, the Fragility Index is a calculation that provides the absolute number of patients or events from an RCT whose alternate outcome would have resulted in the study no longer being statistically significant. Web-based Fragility Index calculators are now readily available [9]. The Fragility Index complements the *p*-value and confidence intervals, and may help clinicians to identify how easily a particular RCTs statistical significance may be overturned.

Recent data from adult RCTs showed that statistically significant outcomes were often contingent on only a small number of patients and were thus statistically fragile [10,11,12]. To date, there have been no studies evaluating the statistical fragility of pediatric RCTs. The purpose of this pilot study was to assess the feasibility of performing a large-scale analysis of fragility in pediatric RCTs.

## 2. Methods

A literature search using OVID Medline and PubMed was executed to identify pediatric RCTs with human subjects, aged 0–18 years, performed between 2000 and 2015. Additional restrictions to focus the cohort on clinically impactful outcomes were made with keyword and MeSH terms including critical care, intensive care, and mortality. English-language abstracts were then screened for inclusion. A convenience sample was generated by restricting results to available English-language studies published in peer-reviewed medical journals with subjectively high impact factors. Studies were included if they were RCTs with statistically positive findings and in which there was an explicitly stated sample size and power calculation with a dichotomous primary outcome between two randomized parallel groups without crossover.

Investigators independently extracted data from each trial. Data elements included the overall trial outcome, number of patients randomized, number of patients analyzed, and number of patients who experienced an outcome in the intervention, as well as control groups, *p*-value, and number of patients who were lost to follow-up. For trials with multiple reported outcomes, only the stated primary outcome was analyzed for fragility. The results of each RCT were extracted and represented in a two-by-two contingency table. As previously described by Walsh et al., in the intervention group, the Fragility Index was calculated by moving a subject from the undesired outcome to the desired outcome, while maintaining the intervention group sample size and then recalculating the two-sided *p*-value for Fisher’s exact test [10]. Events were sequentially added until the calculated *p*-value became equal to or greater than 0.05. The number of new events required to achieve a *p*-value that was no longer significant was designated the Fragility Index for that trial. Characteristics of sampled studies were summarized using descriptive statistics. The Fragility Index was compared to RCT sample size and to the number of study intervention events, and correlations were assessed using a Pearson’s Correlation Coefficient and two-tailed *t*-test (IBM SPSS Statistics for Windows, Version 21.0., Armonk, NY, USA).

## 3. Results

A total of 429 abstracts were screened for inclusion. After applying inclusion and exclusion criteria and assessing for journal quality, 17 RCTs underwent Fragility Index analysis (Table 1) [13,14,15,16,17,18,19,20,21,22,23,24,25,26,27,28,29].

**Figure 1:**
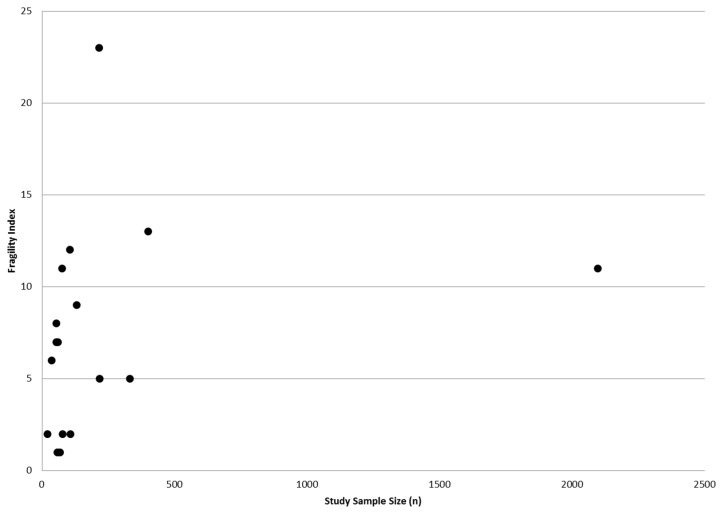
Correlation between RCT sample size and calculated Fragility Index (*r* = 0.249, *p* = 0.335).

The median number of patients in the analyzed RCTs was 152 (range = 41−3141) and the median number of intervention events was 19 (range = 3–221). The spread of original *p*-values was 0.0001–0.04. Statistical significance of *p* < 0.01 was found in 65% (11/17) of the RCTs, and 29% (5/17) had statistical significance of *p* < 0.001. None of the trials were stopped early. The median Fragility Index was 7 (range = 2–23) with an interquartile range of 2–11. In 41% (7/17) of the studies, the number of patients lost to follow-up or withdrawn prior to analysis was equal to or greater than the Fragility Index. There was no correlation between the RCT sample size and the Fragility Index (*r* = 0.249, *p* = 0.335) (Figure 1).

**Figure 2:**
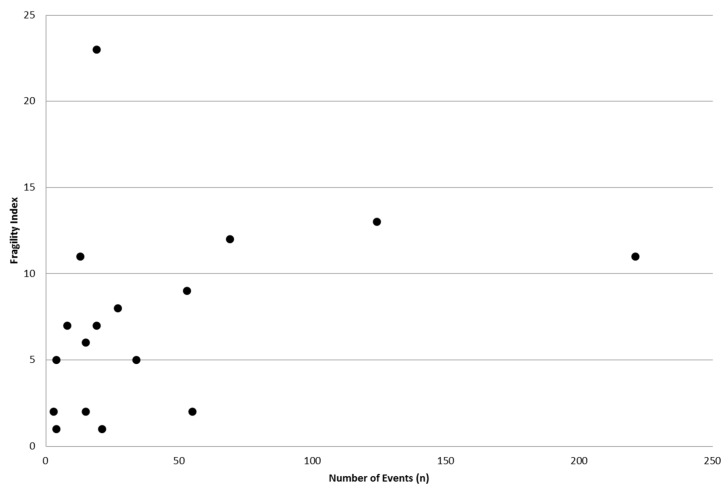
Correlation between RCT event number and calculated Fragility Index (*r* = 0.250, *p* = 0.334).

Similarly, there was no correlation between the size of the event group and the Fragility Index (*r* = 0.250, *p* = 0.334) (Figure 2).

**Figure 3:**
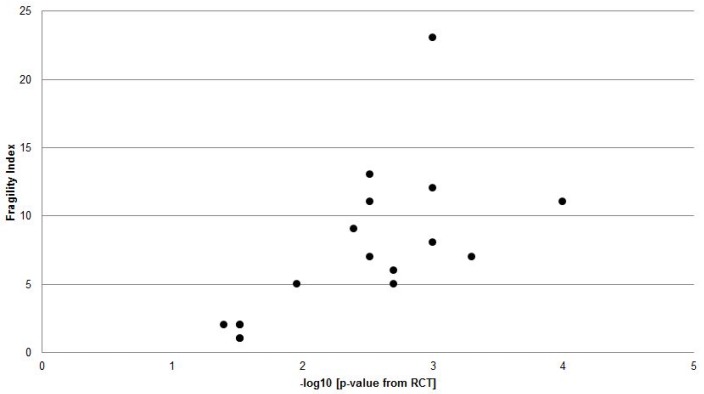
Correlation between negative Log RCT *p*-value for primary outcome and calculated Fragility Index (*r* = 0.700, *p* = 0.002).

However, there was a strong negative correlation between the RCT *p*-value and the Fragility Index (Figure 3).

By including data from the study by Maitland et al., a large skew in both sample size and event size was noted. Statistical analysis was subsequently performed excluding these data to assess for the impact of this RCT on the outcomes of the Fragility Index analysis. As with the primary analysis, there was no correlation between the RCT sample size and the Fragility Index (*r* = 0.314, *p* = 0.236), or between the event size and the Fragility Index (*r* = 0.405, *p* = 0.120) once these data were excluded. Additionally, the negative correlation between the *p*-value and the Fragility Index remained significant, however, the correlation was weaker (*r* = −0.583, *p* = 0.003).

## 4. Discussion

This study demonstrates that statistically significant results from pediatric critical care RCTs with dichotomous outcomes frequently hinge on 7 or fewer actual patient events. Moreover, 25% of pediatric RCTs with a sample size and power calculations indicating an appropriate study design demonstrated that a different outcome for as few as two patients would have resulted in the loss of statistical significance for the RCT primary outcome. RCTs with fragile results were found across a wide range of sample sizes, and larger studies did not necessarily result in larger Fragility Indices. Additionally, in nearly half of the RCTs studied, more participants were excluded from analysis than would be required to make the results of that RCT no longer statistically significant. An RCT with a very small Fragility Index and one where the Fragility Index is smaller than the number of patients not analyzed put those RCT findings at high risk for loss of significance if the study were to be repeated.

The outcomes of any RCT require a clinician to apply clinical judgement to the findings prior to imposing the results on patients. Although clinical trial outcomes may result in statistical significance, namely by assigned *p*-values and confidence intervals, clinical significance may be absent. Paired with a Fragility Index, additional qualitative statistical measures including number needed to treat (NNT) and confidence intervals may offer clinicians additional insights into both the reliability and clinical applicability of the RCT results. The Fragility Index is the only statistic that can provide a reader with an objective measure of exactly how many patients would be required to make the RCT findings no longer statistically significant. Studies with large Fragility Indices indicate that a large number of patients would have had to have experienced an alternate outcome before the significant findings would have been reversed. Alternatively, a study with a very small Fragility Index suggests a high probability that, if repeated, the statistically significant outcome of that RCT may be different. In the present study, the median number of patients whose alternate outcome would convert a significant study to one with non-significant findings was 7, which should give clinicians pause when applying the results of those particular studies to their own patient care. It is important to note that the more significant the RCT study outcome, as indicated by a smaller *p*-value, the larger the Fragility Index, suggesting that with higher levels of significance, there is less fragility and a lower chance of subsequent studies resulting in a non-significant outcome.

The presentation of a Fragility Index in isolation provides very limited value. For example, clinicians may assign different clinical relevancy to a Fragility Index of 3 if the sample size was 30, compared to the same Fragility Index where the sample size was 300. That there was no correlation between sample size and Fragility Index is counter to the usual thought that larger sample sizes will somehow ensure reliability in the statistical significance of a particular RCT. Additionally, clinicians should be concerned that in spite of adequate power and sample size calculations, a quarter of the RCTs in this study had more patients lost to follow-up than would have been required to convert a statistically significant outcome to one of non-significance. The routine calculation and publication of the Fragility Index may better allow clinicians to assess and interpret the findings of a particular RCT.

There are a number of limitations to this study. First, this study was conducted with a convenience sample of RCTs from peer-reviewed medical journals with high impact factors. The theme of critical care was specifically chosen to try to narrow the scope of the pilot data. There are likely many more RCTs from less-read or infrequently cited medical journals that were overlooked in this study. Also, there are likely additional studies outside of the critical care themes that could have been applied to this trial. However, comparing the number of eligible trials to the number of abstracts screened, the data in this study reveal a similar ratio to the larger trials published in the adult literature. Additionally, the RCT by Maitland et al. could have influenced the overall outcomes of this study, given the relatively larger sample size, and skew to the data compared to the other included trials. However, the secondary analysis did not reveal a meaningful change in the correlations between Fragility Index and sample size, event size, or *p*-value once this trial was eliminated. Another limitation is that only those studies in which the primary stated outcome was dichotomous were included in the Fragility Index calculations. Continuous outcome variables do not readily lend themselves to calculation of a Fragility Index, and as such, clinically meaningful studies with continuous outcome measures were excluded from this analysis. In order to calculate a Fragility Index on results with continuous outcome variables, those outcomes must first be dichotomized around an arbitrary set-point which was not attempted in this pilot study. Furthermore, only studies in which the primary dichotomous outcome was statistically significant in a positive or clinically meaningful direction were included. Negative studies do not lend themselves to assignment of a Fragility Index; however, one could postulate that a similar measure may add value to such studies.

## 5. Conclusions

Pediatric RCTs with significant findings can be statistically fragile. Adding the Fragility Index calculation, along with *p*-values and confidence intervals, may enable clinicians to make more informed decisions regarding the clinical applicability and stability of published RCT outcomes. A Fragility Index is an easily calculated metric that may assist in applying clinical relevance to statistically significant outcomes in pediatric RCTs with dichotomous outcomes.

## Figures and Tables

**Table 1:** Summary of included RCTs and extracted data elements.

Lead Author	Sample Size	Intervention Group Size	Intervention Events	Control Events	Fragility Index
Christou H., et al.	41	21	3	11	2
Kicklighter S.D., et al.	103	53	8	23	7
Willson D.F., et al.	152	77	15	27	2
Manzoni P., et al.	322	216	19	31	23
Yeh T.F., et al.	116	60	19	34	7
Lin H.C., et al.	434	217	4	20	5
Simbruner G., et al.	111	53	27	48	8
Jacobs S.E., et al.	208	107	55	67	2
Maitland K., et al	3141	2097	221	76	11
Choong K., et al.	258	130	53	29	9
Jack T., et al.	807	401	124	166	13
Bhatnagar S., et al.	680	332	34	55	5
McCarthy L.K., et al.	72	37	15	27	6
Kumar S., et al.	135	67	21	34	1
Ventura A.M., et al.	120	57	4	17	1
Banupriya B., et al.	150	75	13	36	11
O’Shea J.E., et al.	206	104	69	42	12
